# Radiofrequency ablation of a misdiagnosed Brodie’s abscess

**DOI:** 10.2349/biij.7.2.e17

**Published:** 2011-04-01

**Authors:** RS Chan, BJJ Abdullah, S Aik, CH Tok

**Affiliations:** 1 Department of Biomedical Imaging, Faculty of Medicine, University of Malaya, Kuala Lumpur, Malaysia; 2 Department of Orthopaedic Surgery, Faculty of Medicine, University of Malaya, Kuala Lumpur, Malaysia

**Keywords:** Brodie’s abscess, osteoid osteoma, radiofrequency ablation, misdiagnosis

## Abstract

Radiofrequency ablation (RFA) therapy is recognised as a safe and effective treatment option for osteoid osteoma. This case report describes a 27-year-old man who underwent computed tomography (CT)-guided percutaneous RFA for a femoral osteoid osteoma, which was diagnosed based on his clinical presentation and CT findings. The patient developed worsening symptoms complicated by osteomyelitis after the procedure. His clinical progression and subsequent MRI findings had led to a revised diagnosis of a Brodie’s abscess, which was further supported by the eventual resolution of his symptoms following a combination of antibiotics treatment and surgical irrigations. This case report illustrates the unusual MRI features of osteomyelitis mimicking soft tissue tumours following RFA of a misdiagnosed Brodie’s abscess and highlights the importance of a confirmatory histopathological diagnosis for an osteoid osteoma prior to treatment.

## INTRODUCTION

Brodie’s abscess, a localised subacute or chronic osteomyelitis [1] is a great mimicker of bone tumours [2]. The clinical and radiological presentation of Brodie’s abscess can be very similar to osteoid osteoma. This case report describes a patient who had a Brodie’s abscess in the femoral diaphysis, which simulated an osteoid osteoma both clinically and radiologically. The patient underwent radiofrequency ablation (RFA) therapy, which resulted in unusual MRI features of osteomyelitis mimicking soft tissue tumour. In retrospect, a confirmatory histopathological diagnosis should have been obtained before treatment was initiated.

## CASE REPORT

**Figure 1:**
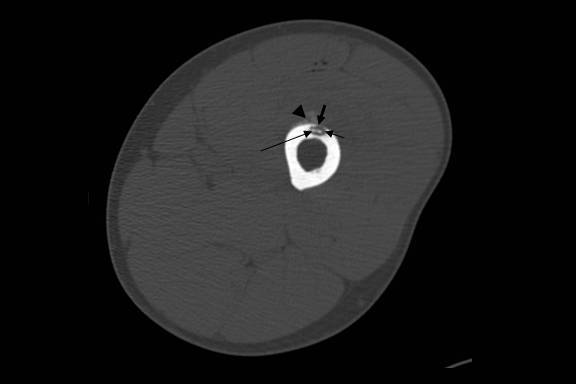
CT image in bone window showing a lucent cortical lesion. Retrospectively, the central calcification (long thin arrow) and the inner margin of the nidus (short thin arrow) were irregular. There was periosteal reaction (arrow head) with a small cortical break (short fat arrow).

A 27-year-old previously healthy man presented with one-month history of left thigh pain which was worse at night. There was deep tenderness at his upper left thigh but no swelling or skin changes. Computed tomography (CT) revealed a small calcified nidus surrounded by a radiolucent rim within the thickened anterior cortex in the shaft of the left femur, with periosteal reaction (Figure 1). The patient underwent CT-guided percutaneous RFA with a presumptive diagnosis of osteoid osteoma. He was symptom-free after the procedure. The histopathological examination found scanty fibrous tissue and bone with infiltration by inflammatory cells. However, a culture of the specimen was not done.

**Figure 2:**
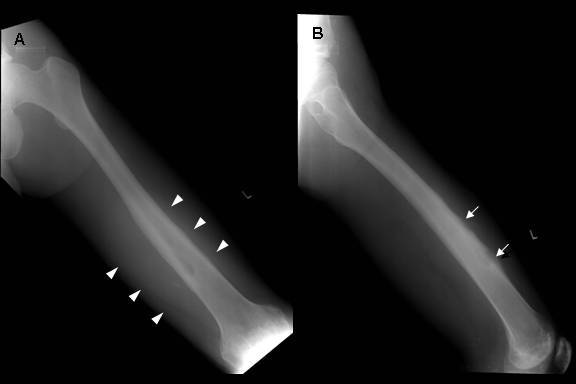
Radiographs of the left femur after RFA demonstrated periosteal reactions (white arrows) (B) with adjacent soft tissue swelling (arrow heads) (A) over the site of previous RFA.

**Figure 3:**
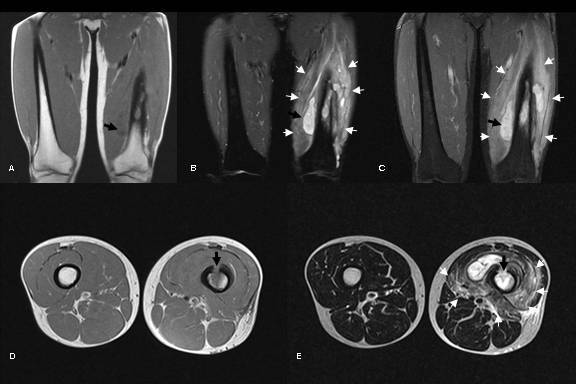
Coronal images showing mass-like lesions (black arrows) along left femoral shaft which were isointense to skeletal muscles on T1-weighted images (A), hyperintense on STIR images (B) and homogenously enhancing post-gadolinium (C). Axial T1-weighted images (D) and T2-weighted images (E) showing thickened anterior cortex with a focal break forming a cloaca (black arrows). Ill-defined areas of high signal changes on T2-weighted, STIR and post-gadolinium images in the marrow cavity at previous RFA site and in the adjacent muscles reflects inflammation and oedema.

Three weeks later, he developed progressive left thigh pain with erythema and induration over the quadriceps despite anti-inflammatory medications and physiotherapy. Radiographs demonstrated periosteal reaction over the site of previous RFA (Figure 2), which raised the clinical suspicion of osteomyelitis. MRI of his left thigh demonstrated thickened anterior cortex with a cloaca and inflammatory changes in the adjacent marrow and skeletal muscles. There were several well-circumscribed, enhancing mass-like lesions beneath the deep muscles adjacent to the site of previous RFA (Figure 3). A diagnosis of post-RFA osteomyelitis was made, with a differential diagnosis of intramuscular soft tissue tumour.

He underwent an open biopsy and surgical irrigation. Pockets of blood-stained soft tissue were discovered along the femoral shaft. These corresponded to the intramuscular masses seen on MRI and were proven to be granulation tissue on histopathological examination. There was inflammation of the adjacent muscles but no abscess collection. His symptoms improved following the surgical irrigation and oral Cloxacillin therapy. Over the subsequent months, he developed several episodes of recurrent left thigh swelling with purulent sinus discharge and was treated with multiple courses of oral antibiotics. His symptoms eventually resolved after a second surgical irrigation and an extended course of Cefoperazone.

**Figure 4:**
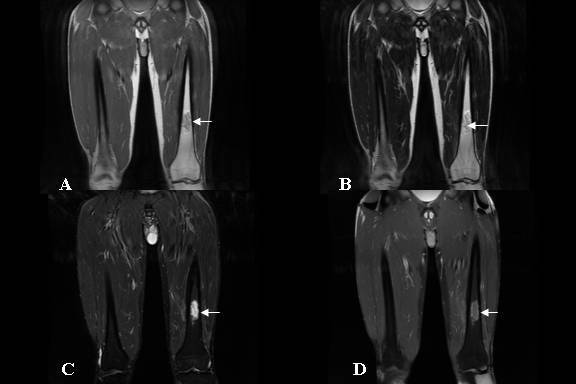
Coronal images after second surgical irrigation and completed antibiotic treatment showing resolution of the soft tissue masses. The small area of residual abnormal marrow signal (arrow) which was hypointense on T1-weighted images (A), hyperintense on T2 weighted (B), STIR (C) and post-gadolinium (D) images was consistent with residual marrow oedema.

Follow-up MRI performed four months after the second surgery demonstrated a small area of low T1signal, high T2 and high STIR signal within the marrow cavity, which was enhanced post-gadolinium, and was consistent with residual marrow oedema (Figure 4). There was complete disappearance of the mass lesions and deep muscle enhancement which were demonstrated in the earlier MRI. The patient has remained well eight months after the second surgery.

## DISCUSSION

Osteomyelitis is known as the great masquerader. It mimics various benign and malignant conditions, especially in the subacute state. Up to 90% of cases are initially misdiagnosed, with a mean delay of 3 months to the correct diagnosis, with 50% of them wrongly diagnosed as tumours [3].

First described by Sir Benjamin Brodie in 1832 [3], Brodies’s abscess is a localised form of subacute or chronic osteomyelitis which may be clinically and radiologically indistinguishable from other infective or neoplastic diseases [2, 3]. Its formation is postulated to be due to the combination of a good host-resistance with a low virulence of the infective organism (which is most commonly Staphylococcus aureus) [2, 3]. Occurring predominantly in young adult males with a history of trauma, recurrent pain and localised tenderness are common presenting symptoms, and are usually associated with fever, chills or malaise due to systemic infection. However, in a significant number of patients, localised pain or vague symptoms ranging from 1 month to 3 years are the only manifestations of a Brodie’s abscess [2]. Clinically, a Brodie’s abscess is indistinguishable from an osteoid osteoma.

Radiographically, Brodie’s abscess is typically a lucent medullary lesion varying in size from less than 1 cm to more than 4 cm in diameter with marginal sclerosis and cortical thickening in the diaphysis or metaphysis of the long bones of the lower extremities [1]. A sinus tract may not be obvious radiographically but is often demonstrated on CT. In contrast, osteoid osteoma is characterised by a round, lucent nidus which is smaller than 15 mm, with surrounding sclerosis and within the thickened cortex of a long bone [4, 5].

A wide range of anatomical and radiographic variation has been described in both Brodie’s abscess and osteoid osteoma [5], with many similarities between the two, making them indistinguishable from each other. A sequestrum in Brodie’s abscess may simulate the nidus of an osteoid osteoma, as in the case described in this report. A few distinguishing imaging features have been described, though these are not pathognomonic. A serpentine sinus tract, a cloaca or a soft tissue mass is suggestive of a Brodie’s abscess. According to Mahboubi, a central, round, smoothly-marginated calcification within a nidus of smooth inner margin is suggestive of an osteoid osteoma. In contrast, the sequestrum of osteomyelitis is often eccentric and is within an irregularly-marginated lucency [3].

Gledhill and Roberts *et al.* classified Brodie’s abscess based on its anatomic location and morphology which simulates other lesions [1, 3]. A type I Brodie’s abscess is a solitary metaphyseal lucency surrounded by sclerosis which may simulate eosinophilic granuloma [1]. A type II Brodie’s abscess is a metaphyseal lucency with cortical erosion resembling an osteosarcoma. A type III Brodie’s abscess is a diaphyseal cortical lucency with periosteal and endosteal reaction, mimicking an osteoid osteoma. A type IV Brodie’s abscess is a diaphyseal lesion with onion-skin periosteal reaction resembling an Ewing’s sarcoma [1]. A type V Brodie’s abscess is a central epiphyseal lucent lesion simulating a chondroblastoma or chondromyxoid fibroma. A type VI Brodie’s abscess is an erosive vertebral lesion mimicking tuberculosis [3]. The imaging appearance in this case fits into a type III Brodie’s abscess which resembles an osteoid osteoma.

This patient did not have an MRI prior to RFA. MRI can be of use in distinguishing tumour from infection. Grey *et al.* described an enhancing penumbra of marginally higher signal intensity than the abscess cavity but of lower signal intensity than the fatty marrow on T1-weighted images of a Brodie’s abscess [3]. A “target appearance of four concentric layers consisting of central necrosis surrounded by granulation tissue, a third layer of sclerosis and an outermost rim of oedema” has also been described [1, 2]. From the centre to the periphery, these appear as low, intermediate, very low and low signal intensity on T1-weighted images; and high, intermediate, low and high signal intensity on T2 weighted images [2]. These MRI appearances of osteoid osteoma are non-specific and can be misleading [3, 5]. The nidus of an osteoid osteoma appears isointense to skeletal muscle on T1-weighted images, hyperintense on T2-weighted images and is not enhanced with Gadolinium [5]. In some cases there may be extensive marrow and soft tissue changes [3].

The treatment for a Brodie’s abscess and osteoid osteoma is different. For Brodie’s abscess, surgery often offers rapid symptom relief, as in this patient [2]. For osteoid osteoma, CT-guided percutaneous RFA has consistently been proven to be the treatment of choice because of its high success rate and low complication rate [4].

A diagnosis of osteoid osteoma was made in this patient based on the age of the patient, symptoms and imaging features of the lesion, the absence of a history of trauma, local or systemic symptoms or signs of infection [3].

Distinguishing neoplasm from osteomyelitis in general can be challenging. Osseous destruction, aggressive periosteal reaction and enhancing intra-osseous or extra-osseous granulation tissue may be demonstrated in both. In osteomyelitis, cortical destruction tends to be more focal and is commonly associated with exuberant and infiltrative soft tissue enhancement which represents oedema and granulation tissue. In bone tumour, cortical destruction tends to be more diffuse and is often associated with less dominant soft tissue oedema. The appearance of intra-osseous or juxta-cortical soft tissue abscesses, though not seen in all, are almost diagnostic of an infective process. In the absence of a pathological fracture, loculated fluid collections are unusual in bone tumour [6].

The differential diagnoses of intramuscular mass lesions on MRI include abscess, haematoma, tumour, myositis ossifican, sarcoidosis, parasitic infection and focal myositis [7]. Granulation tissues are typically isointense on T1-weighted images, hyperintense on T2-weighted images and enhancing, and are associated with infiltrative inflammatory oedema [6]. The appearance of discrete masses of granulation tissue mimicking tumour in this case is unusual and may be attributed to RFA of a Brodie’s abscess causing flare-up of the inflammatory reactions.

## CONCLUSION

In summary, this case report illustrated a young man who was suffering from a type III Brodie’s abscess in the left femoral diaphysis which was misdiagnosed as an osteoid osteoma and underwent RFA which resulted in an unusual MRI appearance of osteomyelitis, with well-circumscribed, mass-like granulation tissue. This case highlighted the importance of a confirmatory histopathological diagnosis in cases of clinically and radiologically suspected osteoid osteoma prior to the initiation of treatment. As the old dictum emphasises “culture every tumour, biopsy every infection” [3], a wrong diagnosis may lead to inappropriate treatment, prolonged patient’s symptoms and additional costs.
